# A case of resistance to thyroid hormone beta and literature review: Case report

**DOI:** 10.1097/MD.0000000000048504

**Published:** 2026-05-01

**Authors:** Zirao Yang, Shuang Ma, Yuehua Dong, Xi Tian, He Wang

**Affiliations:** a Department of Endocrinology, Baoding No.1 Central Hospital, Baoding, Hebei, China.

**Keywords:** differential diagnosis, gene mutation, genetic testing, intellectual disability, thyroid hormone receptor, thyroid hormone resistance syndrome

## Abstract

**Rationale:**

Resistance to thyroid hormone (RTH) syndrome is an uncommon disorder of thyroid function that is frequently misdiagnosed or overlooked clinically. We identified a heterozygous mutation in the *THRβ* gene (c.1357C > A [p.Pro453Thr]) in a patient with resistance to thyroid hormone beta. This locus variation has rarely been reported domestically or internationally.

**Patient concerns:**

The patient, a 13-year-old female presenting with goiter, was found to have persistent high free triiodothyronine and free thyroxine levels with a non-suppressed thyroid-stimulating hormone in the absence of classic hyperthyroid symptoms.

**Diagnoses:**

Based on the patient’s history, physical examination, imaging studies, and genetic testing, the diagnosis of resistance to thyroid hormone was definitively established.

**Interventions:**

The medication was discontinued based on the patient’s clinical status. The management plan was transitioned to a strategy of watchful waiting, with scheduled follow-ups to monitor the patient’s status.

**Outcomes:**

Following a genetic diagnosis, the patient has been followed for 24 months under an active surveillance strategy, which includes annual thyroid function tests.

**Lessons:**

Enhanced clinical vigilance is imperative to mitigate diagnostic bias and errors associated with this condition, thereby ensuring timely and accurate diagnosis and appropriate therapeutic intervention.

## 1. Introduction

Resistance to thyroid hormone (RTH) syndrome is a syndrome of dysfunction in thyroid hormone (TH) synthesis, activation, transport, and receptor-dependent trans-activation that occurs in familial and sporadic cases. The estimated incidence ranges between 1/40,000 and 1/50,000.^[1,2]^ The thyroid hormone receptor (TR) has 2 subtypes, TRα and TRβ, encoded by the *THRα* and *THRβ* genes on chromosomes 17 and 3, respectively.^[3]^ Eighty-five percent of RTH cases are associated with mutations in the *THRβ* gene, and only a small number of RTH cases are associated with mutations in *THRα* or other causes. RTH caused by *THRβ* gene mutation is called β-type thyroid hormone resistance syndrome (resistance to thyroid hormone β [RTHβ]), and is mostly a chromosomal dominant inherited disease. The main characteristics of RTHβ are free thyroxin (FT4) and free triiodothyronine (FT3). Elevated levels of FT3 and normal or mildly elevated levels of thyroid-stimulating hormone (TSH).^[4]^ The clinical phenotypic heterogeneity of RTHβ patients is large, which may be related to the type and site of TRβ mutation and the co-regulatory factors of TRβ. To date, several hundred cases of RTH have been reported in the literature, involving approximately 170 distinct pathogenic mutations in the *THRβ* gene.^[5]^ Individuals with RTHβ typically maintain an almost normal metabolic state. However, their clinical presentation is complex. Due to variations in tissue responsiveness to TH, symptoms characteristic of both hormone deficiency and excess can coexist. The most frequent manifestations are tachycardia and goiter.^[6]^ In addition, some cases may present with a variety of less common features, such as short stature, intellectual developmental delay, growth retardation, and recurrent otorhinolaryngological infections.

In this case, the patient showed neck thickening, and the TSH was normal or slightly elevated, while triiodothyronine (T3) and thyroxine (T4) were elevated. Thyrotropin receptor antibody and antithyroid peroxidase antibodies were increased. Thyroid overall iodine uptake was enhanced. The ultrasonography of thyroid indicated heterogeneous enlargement of thyroid and cystic nodules in the left lobe of thyroid. There was no occupation in the saddle area. Genetic testing revealed that the patient had A heterozygous mutation of the *THRβ* gene, the missense variant c.1357C > A(p.Pro453Thr), which was defined as a pathogenic variant and confirmed the diagnosis of RTHβ.

## 2. Case presentation

### 2.1. Patient information and clinical findings

The patient, a 13-year-old female, was admitted to the Department of Endocrinology of the First Central Hospital of Baoding City in February 2024 due to “1.5 years of neck thickening,” total thyroxine (TT4), FT3, and FT4 were elevated and TSH was normal, and TT4 was normal after thyroxine tests in other hospitals in 2022. The patient was diagnosed with “hyperthyroidism.” “Thyroidine” (the main ingredient is methimazole) was treated, and the drug was stopped automatically after 2 months of use; in January 2024, the thyroid function was reexamined in the other hospital again, indicating that TSH was slightly higher, FT3 and FT4 were increased, and the patient was still diagnosed as “hyperthyroidism” and was given “methimazole” treatment (see Table 1 for details). Physical examination upon admission revealed height 153.7 cm (25th–50th percentile of children of the same age and sex), weight 37 kg (3rd–10th percentile of children of the same age and sex), normal face, no exophthalmos, bilateral 3rd degree thyroid swelling, firm in consistency, no tenderness, normal range of motion, no nodules, fine tremors negative in the fingers, fine hair visible on the lower abdomen and back. Pubic hair Tanner stage 4. Auxiliary examination results: blood routine, liver and kidney function, electrolyte, heart color ultrasound, abdominal color ultrasound, saddle area nuclear magnetic no obvious abnormality. Cortisol (8:00) 219.00 ng/mL (57.20–194.20 ng/mL), adrenocorticotropic hormone (8:00) 38.10 pg/mL (6–48.00 pg/mL), growth hormone 12.80 ng/mL↑ (0–10 ng/mL), 25-hydroxyvitamin D: 11.30 ng/mL (30–70 ng/mL), total type I collagen ammonia terminal prolongating peptide: 141.30 ng/mL (9.06–76.24 ng/mL), β-collagen special sequence: 0.94 ng/mL (0–50 years old: <0.584 ng/mL); insulin-like growth factor 1: 260.00 ng/mL (80–900 ng/mL); total triiodothyronine: 3.56 nmol/L↑ (1.3–3.1 nmol/L), TT4: 220.00 nmol/L↑ (66–181 nmol/L), TSH: 4.51 μIU/mL↑ (0.27–4.2 μIU/mL), FT3: 10.30 pmol/L↑ (3.1–6.8 pmol/L), FT4: 41.70 pmol/L↑ (12–22 pmol/L); serum trans-triiodothyronine: 0.73 ng/mL (0.31–0.95 ng/mL); thyroglobulin: 104.00 ng/mL↑ (5–55 ng/mL), thyroid peroxidase antibodies: 883.00 IU/mL↑ (0–30 IU/mL), thyrotropin receptor antibody 1.74 IU/L↑ (0–1.5 IU/L); ultrasonography showed that the size of the left lobe was about 4.6 × 1.9 × 1.6 cm, the size of the right lobe was about 5.2 × 2.2 × 1.6 cm, and the isthmus thickness was about 0.3 cm. The left lobe of the thyroid gland was orderly in shape, and a cystic echo nodule with a size of 0.4 × 0.3 × 0.2 cm was visible in the parenchyma. The shape was orderly, the boundary was clear, and the echo of the remaining parenchyma was uneven. Determination of iodine uptake rate of thyroid: the overall iodine uptake function of thyroid was enhanced. Octreotide sensitivity test: TSH value decreased by 64.4% from baseline value (see Table 2). Notably, the patient displayed no signs or symptoms of clinical thyrotoxicosis – such as heat intolerance, significant weight loss, or tremors – despite the markedly elevated TH levels. This dissonance between the biochemical profile and the clinical presentation is a hallmark of RTHβ and was pivotal in steering the differential diagnosis away from conventional hyperthyroidism.

**Table 1:** Summary of patient encounters, including chief complaints, workup, and treatment.

Time	Clinical stage	Chief complaint	Item	Results	Reference range	Treatment
2022.06.04	Initial visit	Neck thickening	TT3 (ng/mL)	1.47	0.87–1.78	Methimazole
			TT4 (μg/dL)	14.25	6.09–12.23	
			TSH (μIU/mL)	1.78	0.56–5.91	
			FT3 (pg/mL)	5.26	2.5–3.9	
			FT4 (ng/dL)	2.3	0.61–1.12	
2022.06.04	–	–	–	–	–	Withdrawal
2024.01.31	Review	Neck thickening	TSH (μIU/mL)	4.32	0.51–4.30	Methimazole
			FT3 (pmol/L)	9.04	3.93–7.7	
			FT4 (pmol/L)	35.40	12.6–21.0	
2024.02.03	Review	Neck thickening	TT3 (nmol/L)	3.56	1.3–3.1	Withdrawal
			TT4 (nmol/L)	220.00	66–181	
			TSH (μIU/mL)	4.51	0.27–4.2	
			FT3 (pmol/L)	10.30	3.1–6.8	
			FT4 (pmol/L)	41.70	12–22	
2024.02.04	Hospitalization for examination	Neck thickening	MRI of the sella turcica	No occupancy observed		Withdrawal
2024.02.08	Octreotide sensitivity test	Neck thickening	TSH value decreased by 64.4% from baseline value			Withdrawal

FT3 = free triiodothyronine, FT4 = free thyroxine, MRI = magnetic resonance imaging, TSH = thyroid-stimulating hormone, TT3 = total triiodothyronine, TT4 = total thyroxine.

**Table 2:** Octreotide sensitivity test.

	0 h	2 h	4 h	6 h	8 h	12 h	24 h	48 h	72 h	Reference range
TT3 (nmol/L)	3.56↑	3.18↑	3.15↑	2.9	2.72	2.58	2.78	2.57	2.39	1.3–3.1
TT4 (nmol/L)	220↑	203↑	197↑	195↑	186↑	178	196↑	173	172	66–181
TSH (μIU/mL)	4.51↑	4.58↑	2.37	1.61	1.8	1.68	2.02	2.64	3.65	0.27–4.2
FT3 (pmol/L)	10.3↑	9.54↑	9.22↑	8.83↑	8.38↑	8.14↑	8.29↑	7.54↑	6.95↑	3.1–6.8
FT4 (pmol/L)	41.7↑	36.1↑	38.2↑	34.9↑	33.9↑	34.6↑	37↑	31.3↑	29.2↑	12–22

FT3 = free triiodothyronine, FT4 = free thyroxine, TSH = thyroid-stimulating hormone, TT3 = total triiodothyronine, TT4 = total thyroxine.

### 2.2. Diagnostic assessment

**Figure 1.**
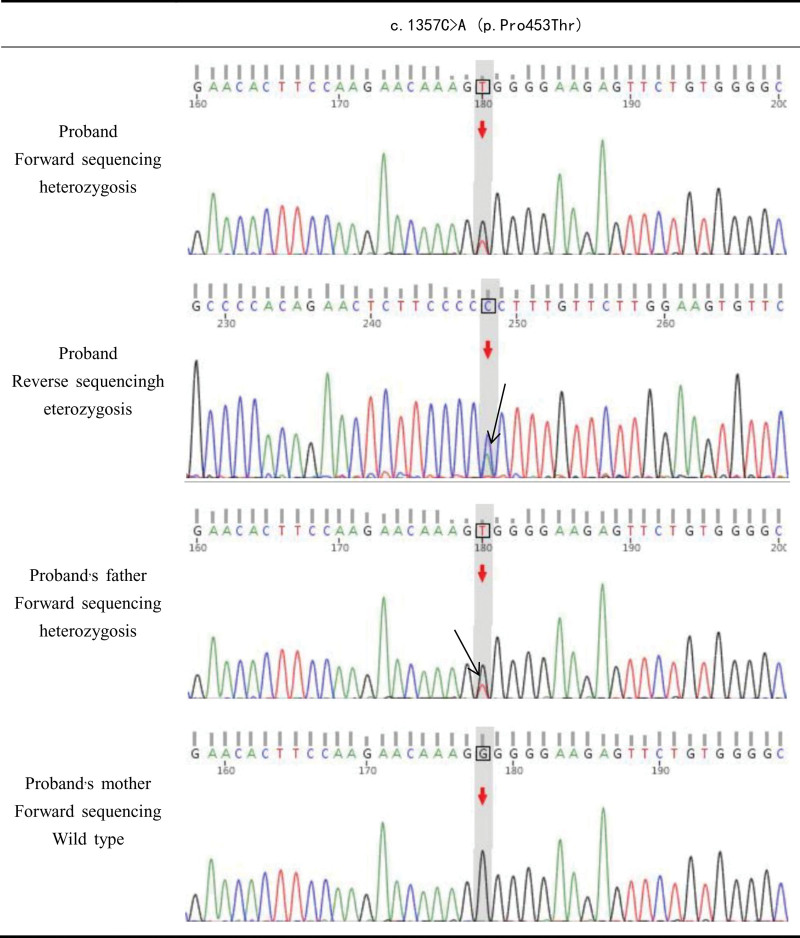
Sanger sequencing of the patient and her parents. Genetic testing revealed a heterozygous mutation in the *RTHB* gene, specifically a nucleotide change at position 1148 from cytosine to adenine, resulting in an amino acid substitution at position 453 from proline to threonine. (Sanger sequencing analysis of the proband revealed a heterozygous mutation. The forward sequence chromatogram showed overlapping peaks of T and G at the same position, indicating the presence of both nucleotides in the template strand. Correspondingly, the reverse sequence chromatogram showed overlapping peaks of C and A. As the wild-type base on the coding strand is C, the concurrent presence of C and A confirms a heterozygous C > A mutation at this locus. Segregation analysis within the family showed that the father carried the same heterozygous C > A mutation, while the mother gene is normal, wild-type, indicating a paternally inherited variant.)

The patient’s initial laboratory workup revealed a characteristic yet paradoxical thyroid profile: significantly elevated serum FT3 and FT4 concentrations alongside a non-suppressed TSH level. In the absence of clinical symptoms of thyrotoxicosis, this biochemical pattern steered the differential diagnosis away from common causes of hyperthyroidism. The possibility of a TSH-secreting pituitary adenoma was subsequently excluded based on the absence of a pituitary mass on sellar magnetic resonance imaging and a negative octreotide suppression test. The convergence of these findings strongly pointed toward a diagnosis of RTHβ. Subsequent genetic analysis identified a heterozygous missense variant in the *THRB* gene, c.1357C > A(p.Pro453Thr), which was determined to be of paternal origin (see Fig. 1). This variant was classified as “Pathogenic” according to the American College of Medical Genetics and Genomics guidelines, supported by the following evidence: PS4 (the mutation is a known recurrent hotspot in RTHβ), PM1 (located in a mutational hotspot and critical functional domain of the ligand-binding pocket), and PM2 (absent from population databases). This genetic confirmation provided the definitive diagnosis.

### 2.3. Therapeutic intervention

This case underscores a critical clinical pitfall. The initial misdiagnosis of conventional hyperthyroidism and the subsequent administration of methimazole represent an ineffective and potentially harmful therapeutic approach. It must be emphatically stated that upon genetic confirmation of RTHβ, all conventional antithyroid modalities – including antithyroid drugs, radioiodine, and thyroid surgery – are contraindicated. Consequently, the cornerstone of management shifts decisively to long-term symptomatic monitoring and the vigilant avoidance of inappropriate treatments.

### 2.4. Follow-up and outcomes

Following a genetic diagnosis, the patient has been followed for 24 months under an active surveillance strategy, which includes annual thyroid function tests. Comprehensive genetic counseling was provided, covering the favorable prognosis, the autosomal dominant inheritance pattern of RTHβ, and the recommendation for first-degree relative screening. The long-term management plan further incorporates annual evaluations of growth and pubertal development, alongside a protocol for periodic neurodevelopmental assessments to address any future concerns. The family expressed that the definitive diagnosis brought significant relief, with the mother noting, “Finally obtaining the genetic diagnosis was a huge relief. It resolved our previous confusion and led to the discontinuation of ineffective medications. We now have a clear plan to support our daughter’s healthy development.”

## 3. Discussion

RTH is a clinical syndrome characterized by reduced sensitivity to THs.^[6]^ In 1967, Refetoff et al^[7]^ first described RTH. Subsequently, in 1988, Usala et al^[8]^ confirmed that RTH is associated with mutations in the TRβ gene. RTHβ is typically categorized into 3 subtypes: generalized RTHβ, pituitary RTHβ, and peripheral RTHβ.^[9]^ In most cases, RTHβ follows an autosomal dominant inheritance pattern. It is well established that RTH is primarily caused by defects in the TR. The molecular structures and sequences of TRα and TRβ proteins are highly similar, with 4 known isoforms: TR-α1, TR-α2, TR-β1, and TR-β2. All isoforms except TR-α2 can bind to TH. These receptor subtypes exhibit distinct tissue distributions. For instance, TRβ2 is predominantly expressed in the pituitary gland and hypothalamus, whereas TRα is mainly expressed in the central nervous system.^[10]^ Consequently, individuals with RTH syndrome may display hyperthyroidism symptoms in some tissues and hypothyroidism symptoms in others, depending on the degree of tissue responsiveness to elevated TH levels.^[11]^

The persistent elevation of free THs (FT3/FT4) with a non-suppressed TSH should immediately raise suspicion for RTH. Failure to recognize this distinct biochemical profile carries a significant risk of iatrogenic harm. As demonstrated in our patient, misdiagnosis as conventional hyperthyroidism can lead to the unnecessary and ineffective use of antithyroid drugs, radioiodine, or surgery. Published case reports^[12]^ have shown that patients presenting with atrial fibrillation were found to have elevated free THs and normal TSH levels, leading to a misdiagnosis of Graves disease and inappropriate treatment with antithyroid medications. The correct diagnosis of RTH was only established after more than 8 years. Another report^[13]^ described a patient with symptoms of cold intolerance and hand pain, who exhibited persistently elevated FT4 and TSH levels and was mistakenly treated with levothyroxine sodium. Ultimately, a *THRβ* gene mutation was identified through genetic testing. The case discussed in this article, while exhibiting different clinical manifestations from the 2 cases mentioned above, similarly involved inappropriate treatment based solely on the patient’s clinical presentation and thyroid function tests. Ultimately, the correct diagnosis of RTHβ was confirmed through genetic testing in all cases, and no further medication or treatment was administered thereafter. Therefore, in such cases, genetic sequencing is no longer optional but is essential, as it provides a definitive diagnosis and prevents years of inappropriate management.

Genetic testing revealed a heterozygous mutation in the *RTHβ* gene, specifically a nucleotide change at position 1148 from cytosine to adenine, resulting in an amino acid substitution at position 453 from proline to threonine, which represents a missense mutation. This locus variation has been a subject of significant interest in international reports. The diagnosis of RTH mandates a systematic differential diagnosis. The initial step is to exclude common thyroid disorders, such as hyper- or hypothyroidism, through standard serological testing. For suspected RTH cases, thyroid function tests and thyroid antibodies should be assayed repeatedly to rule out laboratory interference. Subsequently, conditions that cause abnormal serum TH binding, including familial dysalbuminemic hyperthyroxinemia and abnormalities in thyroxine-binding globulin or transthyretin, must be excluded. A critical step is differentiating RTH from thyrotropin-secreting pituitary adenomas. Both disorders are classified under the Syndrome of Inappropriate Secretion of Thyrotropin, characterized by elevated free TH (FT3/FT4) levels in the presence of non-suppressed TSH. Both RTH and TSH tumors belong to the Syndrome of Inappropriate Secretion of Thyrotropin, characterized by elevated serum FT3 and/or FT4 levels despite normal or elevated TSH levels. Due to the lack of specific symptoms and examination indicators, both conditions are frequently misdiagnosed and mistreated clinically.^[14]^ Therefore, distinguishing between them is essential. Thyrotropin-secreting pituitary adenomas are key factors to consider in the differential diagnosis of RTHβ after excluding interference as the cause of inconsistent thyroid function tests.

Currently, there is no definitive cure for RTH, nor any clear guidelines or expert consensus. Comprehensive examinations should therefore be conducted for newborns with a family history, particularly those exhibiting intellectual disability, deaf-mutism, or abnormal body shapes. Treatment of RTH patients should aim to maintain a normal metabolic state, and most RTH patients can achieve regulation through increased endogenous TH secretion to compensate for tissue insensitivity. A person with hypothyroid or hyperthyroid symptoms may require TH, beta-blockers, or TH analogs.^[15]^ It has been reported that the T3 analogue triiodothyronine can inhibit TSH, relieve goiter, lower TH levels, and improve the symptoms of hyperthyroidism.^[16,17]^ A patient with RTHβ presenting clinical symptoms of hyperthyroidism was treated with T3 analogue triiodothyronine for 5 years, and the symptoms were effectively controlled.^[18]^ While the immediate message is to avoid overtreatment, the real challenge lies in ensuring careful follow-up of growth, puberty, and cognitive function in young patients with RTHβ. Consequently, the cornerstone of managing confirmed RTHβ shifts fundamentally from active intervention to vigilant, proactive surveillance. Our instituted long-term management plan for this young patient includes annual thyroid function testing to establish an individual biochemical baseline; semiannual assessments of linear growth and pubertal development using standardized growth charts and Tanner staging; and periodic neurocognitive and academic evaluations, with a low threshold for formal assessment should any concerns arise from parents or teachers.

## 4. Conclusion

In summary, we identified a heterozygous mutation in the *THRβ* gene (c.1357C > A[p.Pro453Thr]) in a patient with RTHβ. This locus variation has been rarely reported domestically or internationally. Given the high likelihood of misdiagnosis and missed diagnosis in clinical practice, genetic testing plays a crucial role in diagnosing thyroid hormone resistance syndrome. By summarizing and analyzing this rare case and reviewing relevant literature, we aim to enhance disease management, provide clinical guidance, and improve clinician awareness.

## Acknowledgments

The authors thank the patient and her family for their generous participation in this study.

## Author contributions

**Writing – original draft:** Zirao Yang, Shuang Ma, Xi Tian.

**Writing – review & editing:** Zirao Yang, Yuehua Dong, He Wang.
